# ELOVL6 is associated with immunosuppression in lung adenocarcinoma through bioinformatics analysis

**DOI:** 10.1097/MD.0000000000035013

**Published:** 2023-09-08

**Authors:** Binyu Chen, Kaiyu Shen, Tiantian Zhang, Wen-Cang Gao

**Affiliations:** a The Second Clinical Medical College of Zhejiang Chinese Medical University, Hangzhou, China.

**Keywords:** ELOVL6, immune infiltration, information biology, LUAD

## Abstract

The aim of this paper was to reveal the correlation between the expression of ELOVL fatty acid elongase 6 (ELOVL6) gene in lung adenocarcinoma (LUAD) and its clinical significance, immune cell infiltration level and prognosis. Expression profile data of ELOVL6 mRNA were collected from the cancer genome atlas database to analyze the differences in ELOVL6 mRNA expression in LUAD tissues and normal lung tissues, and to analyze the correlation between ELOVL6 and information on clinicopathological features. Based on TIMER database, TISDIB database and GEPIA2 database, the correlation between ELOVL6 expression and tumor immune cell infiltration in LUAD was analyzed. Gene ontology and Kyoto encyclopedia of genes and genomes enrichment analyses of ELOVL6-related co-expressed genes were performed to identify the involved signaling pathways and to construct their co-expressed gene protein interaction networks. Drugs affected by ELOVL6 expression were screened based on the Cell Miner database. These findings suggest that ELOVL6 plays an important role in the course of LUAD, and the expression level of this gene has a close relationship with clinicopathological characteristics and survival prognosis, and has the potential to become a prognostic marker and therapeutic target for LUAD.

## 1. Introduction

Lung cancer is a malignant tumor originating from the mucosa or glands of the trachea and bronchi and is classified into non-small cell lung cancer and small cell lung cancer, which are characterized by high morbidity and mortality.^[1]^ Among them, lung adenocarcinoma (LUAD) is the main histological type of NSCLC, and patients with LUAD have shorter survival time than other types.^[2]^ Due to the insidious onset of LUAD, most patients with LUAD are already at an advanced stage when diagnosed, with a 5-year survival rate of <20%.^[3]^ Despite the current rapid progress in research on chemotherapy, radiotherapy, targeted therapy, and immunotherapy for LUAD, the 5-year survival rate is still relatively low.^[4]^ Therefore, there is an urgent need to identify a new biomarker as a therapeutic target to improve the prognosis of patients with LUAD.

The ELOVL fatty acid elongase 6 (ELOVL6) gene is a member of the ultra-long chain fatty acid elongase family, which catalyzes the elongation of saturated and monounsaturated fatty acids and is mainly involved in fatty acid elongation and lipid acyl CoA biosynthesis.^[5]^ Studies have confirmed that ELOVL6 plays an important role in atherosclerosis,^[6]^ diabetes,^[7]^ fatty liver^[8]^ and other diseases. Saito, R. et al found that ELOVL6 in macrophages may contribute to foam cell formation, while by producing ROS and activating AMPK/KLF4 signaling, it can induce proliferation of aortic smooth muscle cells,^[9]^ leading to the occurrence and progression of atherosclerosis. Removal of ELOVL6 will increase insulin sensitivity and reduce insulin resistance, so that some patients with type 2 diabetes can be cured.^[10]^ More studies have shown that ELOVL6 plays an important role in the occurrence and development of breast cancer,^[11]^ colon cancer,^[12]^ liver cancer,^[13]^ and lung squamous cell carcinoma.^[14]^ However, there are still few reports about ELOVL6 in LUAD. This study aims to explore the expression of ELOVL6 in LUAD and its effect on prognosis and treatment based on multiple databases.

## 2. Methods

### 2.1. ELOVL6 gene data retrieval

This study focused on downloading transcriptomic data of LUAD patients by searching data from the cancer genome atlas (TCGA) database. Transcriptomic data and clinical information from the GSE40791 dataset of LUAD were downloaded from the gene expression omnibus database. The expression data of ELOVL6 in tumor and normal samples were extracted using the “limma” package of R software (version 4.1.0, http://www.r-project.org/) and incorporated into the clinicopathological characterization and survival analysis.

### 2.2. ELOVL6 gene differential analysis and clinicopathological features analysis

The differential expression of ELOVL6 between LUAD tumor samples and normal samples was analyzed in TCGA and gene expression omnibus databases, and the differential expression of the gene was mapped. The relationship between age, gender, pathological stage, T-stage, N-stage, and M-stage and ELOVL6 expression in LUAD patients was then analyzed and mapped using the “ggpubr” package.

### 2.3. Survival prognosis analysis

Based on the Kaplan–Meier plotter database and GEPIA database, the effect of ELOVL6 on the overall survival (OS) of a sample of LUAD patients with complete clinical information in the TCGA database was analyzed. A Cox regression analysis model was developed to carry out univariate and multivariate Cox regression analysis of ELOVL6 expression and clinical data of LUAD patients, taking *P* < .05 as a statistically significant difference, and forest plots were used to present the results.

### 2.4. ELOVL6 co-expressed genes

Using the “limma” package of R software (version 4.1.0, http://www.r-project.org/), we grouped ELOVL6 by median value of expression, and obtained 2 groups of high and low expression, and explored the co-expressed genes of ELOVL6 in 535 LUAD patients in the transcriptome data downloaded from TCGA database, and plotted volcano map, heat map, and gene correlation map.

### 2.5. Gene ontology (GO) analysis

Kyoto Gene and Genome Encyclopedia (KEGG) pathway analysis and gene set enrichment analysis (GSEA) analysis gene ontology (GO), KEGG analysis of ELOVL6 co-expressed genes was performed using the “clusterProfiler” package of R software (version 4.1.0, http://www.r-project.org/) with the setting condition of *P* ≤ .05. Subsequently, the ELOVL6 mRNA data set was divided into high and low ELOVL6 expression subgroups. ELOVL6 mRNA dataset into ELOVL6 high and low expression subgroups. Further, gene enrichment analysis of ELOVL6 was performed using GSEA software (version 4.1.0, http://www.r-project.org/), and the criteria for significant GSEA enrichment were judged as NOM *P* value < .05, and FDR *q*-value < 0.25.

### 2.6. Construction and visualization of PPI protein interactions network

The STRING database was applied to construct protein interaction relationships for the obtained co-expressed genes. Protein interactions between genes were visualized using cytoscap software (version 3.7.2, https://cytoscape.org/) to annotate up- and down-regulated genes.

### 2.7. Correlation of ELOVL6 expression with immune infiltrating cells and immune genes

The TIMER database, TISIDB database, GEPIA2 database and R software (version 4.1.0, http://www.r-project.org/) “reshape2” package and “limma” package were used to investigate the correlation between ELOVL6 expression and immune infiltrating cells and immune gene correlation.

### 2.8. Probing the correlation between ELOVL6 expression and drug sensitivity

Comparisons between 2 groups were performed using the Mann–Whitney U test; comparisons between multiple groups were evaluated using the Kruskal-Wallis H test. ROC curves were plotted using the “pROC” package, the “timeROC” package, the Kaplan–Meier method, and the log-rank method to explore the high and low ELOVL6 The Kaplan–Meier and log-rank methods were used to explore the survival analysis of ELOVL6 high and low expression subgroups. One-way and multi-way cox regression analyses were performed for prognostic factors. *P* < .05 was considered statistically significant.

## 3. Result

### 3.1. Elevated expression levels of ELOVL6 in LUAD

**Figure 1.**
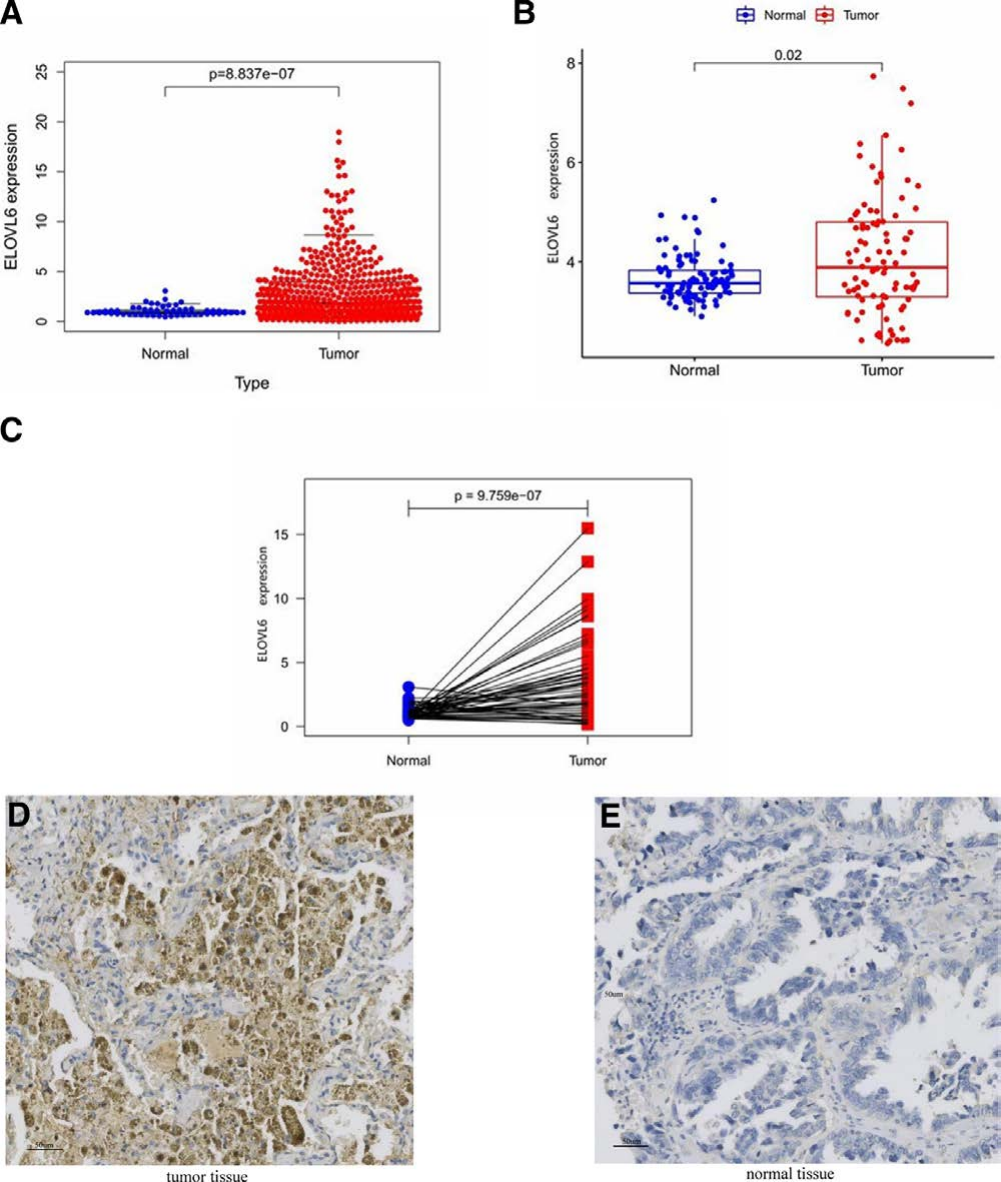
Differential expression of ELOVL fatty acid elongase 6 (ELOVL6) in lung adenocarcinoma (LUAD) and normal lung tissues. (A) Differential expression of ELOVL6 based on the cancer genome atlas (TCGA) database. (B) Differential expression of ELOVL6 based on the gene expression omnibus (GEO) database. (C) Differential expression of ELOVL6 in cancer and paraneoplastic tissues. (D and E) After we analyzed several LUAD tissues, immunohistochemical results showed that ELOVL6 expression was lower in normal lung tissues than in LUAD tissues.

Transcriptomic data obtained from the TCGA database showed that the expression of ELOVL6 was significantly lower in LUAD tissues than in unpaired normal tissues (Fig. 1A). The expression level of ELOVL6 in the GSE40791 dataset was higher in LUAD samples than in unpaired normal samples (Fig. 1B). The differential expression of ELOVL6 in 57 paired cancer tissues versus paraneoplastic tissues from the TCGA database was analyzed, and the results revealed that ELOVL6 expression was significantly lower in paraneoplastic tissues than in cancer tissues (Fig. 1C). These results suggest that ELOVL6 is overexpressed in LUAD tissues.

### 3.2. Correlation of ELOVL6 expression with clinicopathological features in LUAD patients

**Figure 2.**
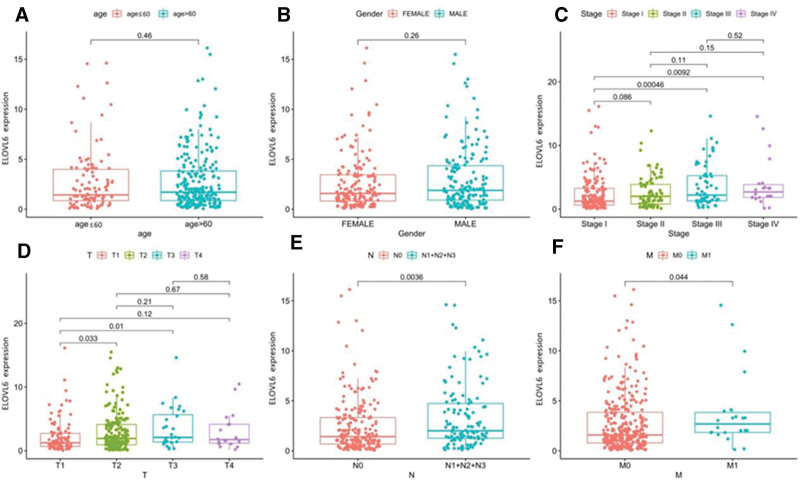
Correlation between ELOVL fatty acid elongase 6 (ELOVL6) expression and clinicopathological features (A–F). ELOVL6 expression increased with increasing pathological TNM stage and was independent of gender and age.

Analysis of the relationship between ELOVL6 expression and clinical data of TCGA revealed that the expression level of ELOVL6 in LUAD correlated with the TNM stage of patients, independent of gender and age. The expression of ELOVL6 increased with increasing stage (Fig. 2).

### 3.3. Correlation between ELOVL6 expression and survival prognosis in LUAD patients

**Figure 3.**
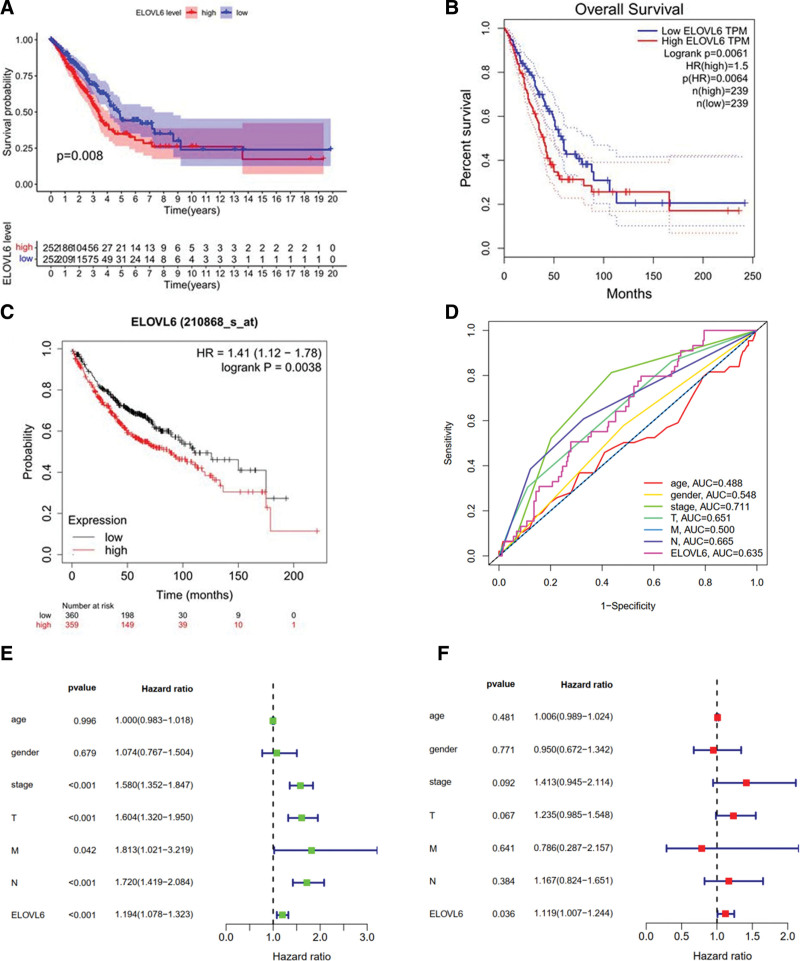
ELOVL fatty acid elongase 6 (ELOVL6) is associated with survival prognosis in lung adenocarcinoma (LUAD) patients. (A–C) Overall survival curves of ELOVL6 in LUAD. (D) Multi-indicator survival ROC curve model for ELOVL6. (E and F) Univariate and multifactorial Cox analysis of ELOVL6 and clinical characteristics.

The results of the survival analysis of ELOVL6 expression and LUAD patients revealed that the OS was significantly longer in the ELOVL6 low expression group than in the ELOVL6 high expression group in LUAD patients, in addition to similar results obtained in the GEPIA database and the Kaplan–Meier plotter database using online analysis tools (Fig. 3A–C). Multi-indicator survival ROC curve model showed AUC values of 0.488, 0.548, 0.711, 0.651, 0.665, 0.500, and 0.635 for age, gender, tumor stage, T stage, N stage, M stage, and ELOVL6, respectively (Fig. 3D). Univariate and multifactorial analyses showed that tumor TNM stage was associated with prognosis of LUAD patients, and ELOVL6 expression was an independent factor for prognosis of LUAD patients (Fig. 3E and F).

### 3.4. Screening of ELOVL6-related co-expressed genes

**Figure 4.**
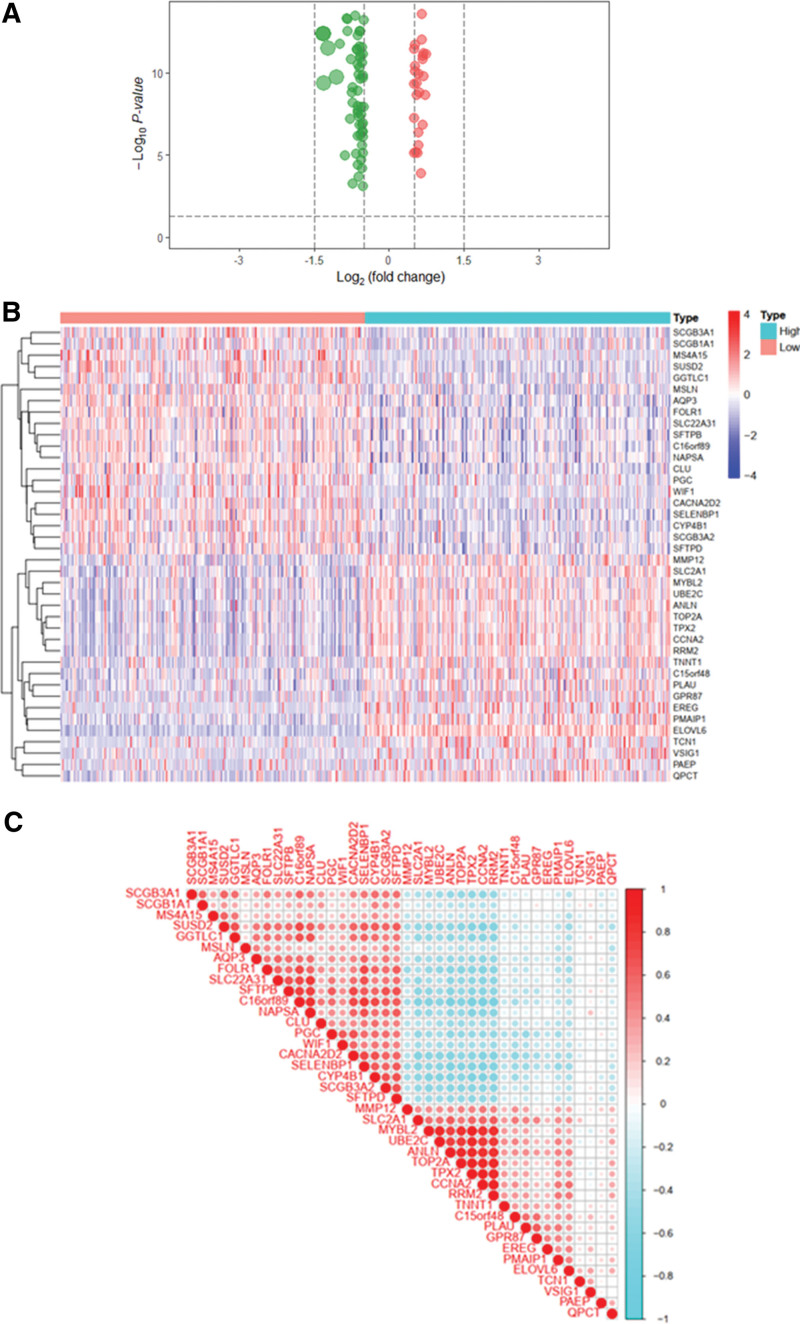
Volcano map, heat map and co-expressed gene correlation map of ELOVL fatty acid elongase 6 (ELOVL6)-related differential genes. (A) Volcano map of co-expressed genes. (B) Heat map of co-expressed genes. (C) Gene correlation map of co-expressed genes.

The 146 co-expressed genes associated with ELOVL6 were screened by R software and plotted on the volcano map (Fig. 4A). Among them, 71 up-regulated co-expressed genes and 75 down-regulated co-expressed genes were included; 20 significantly up-regulated genes and down-regulated genes were selected to draw the co-expressed gene heat map (Fig. 4B), and Spearman correlation coefficients >0, <0, and equal to 0 were indicated in red, blue and white, respectively, in the gene correlation map (Fig. 4C).

### 3.5. GO analysis of ELOVL6-related differential genes, KEGG analysis and GESA analysis of ELOVL6

#### 3.5.1. GO enrichment analysis.

**Figure 5.**
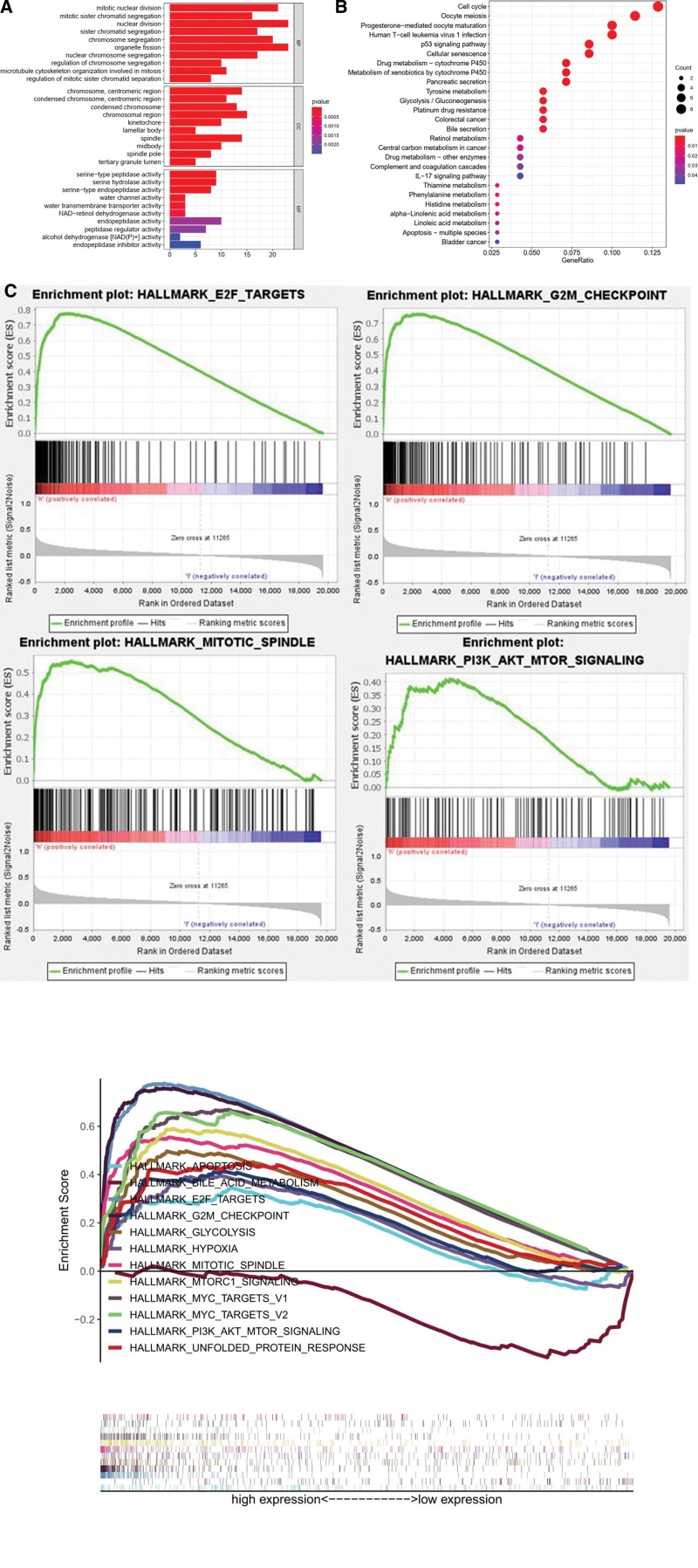
Gene ontology (GO) enrichment analysis, Kyoto encyclopedia of genes and genomes (KEGG) enrichment analysis and gene set enrichment analysis (GSEA) enrichment analysis of ELOVL fatty acid elongase 6 (ELOVL6) co-expressed genes. (A) Enrichment of ELOVL6 co-expressed genes in biological processes (BP), cellular components (CC) and molecular functions (MF). (B) KEGG enrichment analysis of ELOVL6 co-expressed genes. (C and D) Hallmark gene set-based ELOVL6 GSEA enrichment analysis.

We performed enrichment analysis of ELOVL6-related co-expressed genes, and the results suggested that the expression of co-expressed genes was positively correlated with mitotic nuclear division, sister chromatid separation, organelle fission, chromosome trophoblast region, spindle, serine hydrolase activity, peptide chain endonuclease activity, and peptidase regulatory activity (Fig. 5A).

#### 3.5.2. KEGG enrichment analysis.

KEGG enrichment analysis was carried out using R software (version 4.1.0, http://www.r-project.org/) for ELOVL6-related co-expressed genes, and the results suggested that the co-expressed genes were mainly enriched in central carbon metabolism, cell cycle, P53 signaling pathway, cellular senescence, pancreatic secretion, colorectal cancer, and bladder cancer in cancer (Fig. 5B).

#### 3.5.3. GSEA enrichment analysis.

To explore the association of ELOVL6 with Hallmark gene set, the results suggested that E2F signaling pathway, G2M checkpoint, apoptosis, mitotic spindle, MTORC1 signaling pathway, and PI3K- AKT- MTOR signaling pathway were highly active in the ELOVL6 high expression subgroup (Fig. 5C and D).

### 3.6. Construction and analysis of PPI protein interaction network

**Figure 6.**
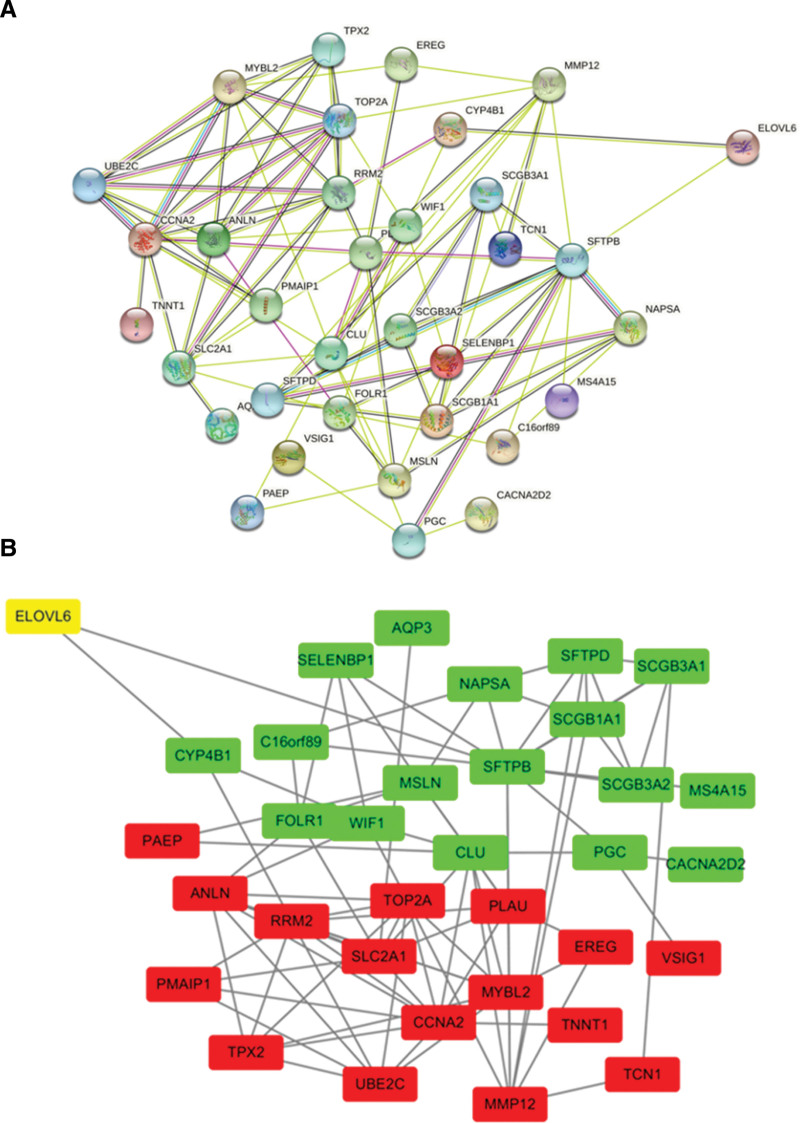
Interprotein interaction network of ELOVL fatty acid elongase 6 (ELOVL6)-related co-expressed genes. (A)Construction of ELOVL6 co-expressed gene network based on STRING database. (B) Visualization of ELOVL6 co-expressed gene network based on cytoscap software (version 3.7.2, https://cytoscape.org/).

The STRING database was used to construct protein interaction networks for the 40 most significantly up- and down-regulated genes associated with ELOVL6 above (Fig. 6A), and genes without protein interaction relationships were screened out. The protein network interactions between co-expressed genes were visualized by Cytoscap software (version 3.7.2, https://cytoscape.org/), and different colors, i.e., red, green and yellow, were used to indicate up-regulated genes, down-regulated genes and ELOVL6, respectively (Fig. 6B).

### 3.7. Correlation of ELOVL6 expression with immune infiltrating cells and immune genes

**Figure 7.**
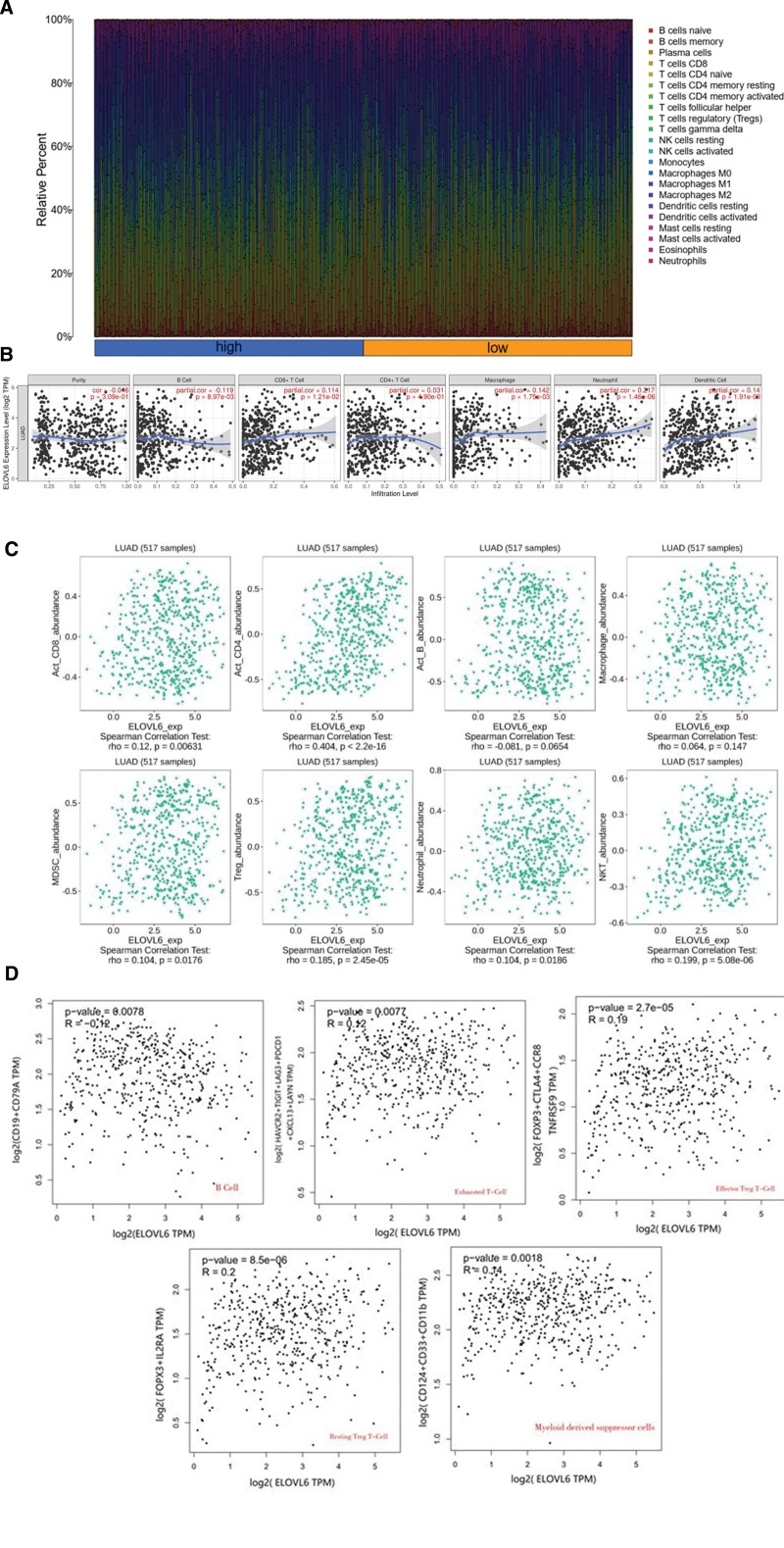
Relationship between ELOVL fatty acid elongase 6 (ELOVL6) and immune cells and immune genes. (A) Histogram of immune cells in different ELOVL6 expression groups. (B) Correlation between ELOVL6 and immune cells based on TIMER database. (C) Correlation between ELOVL6 and immune cells based on TISDIB database. (D) Correlation of ELOVL6 with MDSC and Treg cell marker genes based on GEPIA2 database.

Patients with LUAD were grouped according to the median value of ELOVL6 expression to obtain the immune cell histogram (Fig. 7A); the TIMER database (Fig. 7B) and TISDIB database (Fig. 7C) were used to explore the correlation between ELOVL6 gene and immune cells, and the GEPIA2 database, R software (v4.1.0) were used to explore the association between ELOVL6 and immune marker genes (Fig. 7D), the results showed that in LUAD, ELOVL6 expression was positively correlated with CD8 + T cells, CD4 + T cells, myeloid-derived suppressor cells, regulatory T cells, and neutrophils; and negatively correlated with B cells, and ELOVL6 expression was positively correlated with regulatory T cells and myeloid-derived suppressor cell marker genes and negatively correlated with B cell marker genes (Fig. 7D).

### 3.8. Correlation of ELOVL6 upregulation with drug sensitivity

**Figure 8.**
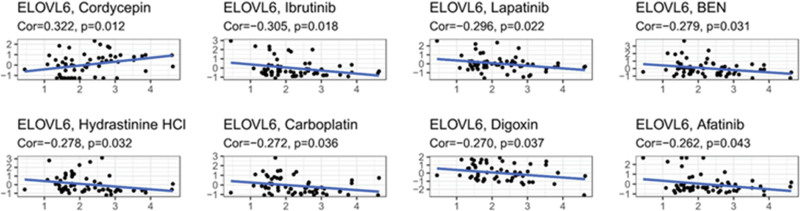
Effect of ELOVL fatty acid elongase 6 (ELOVL6) expression on drug sensitivity.

Exploring the drugs with correlation with ELOVL6 expression, the results suggested that high ELOVL6 expression was negatively correlated with drug sensitivity of lbrutinib, Lapatinib, BEN, Hydrastinine HCI, Carboplatin, Digoxin and Afatinib (Fig. 8).

## 4. Discussion

In recent years, the incidence of LUAD has surpassed that of squamous carcinoma as the most predominant pathological type,^[11]^ accounting for approximately 40% of all lung cancers.^[15]^ However, LUAD has no obvious clinical symptoms in the early stage and is often detected during plain chest radiography, and hematogenous metastasis occurs at an early stage, so the prognosis of patients is relatively poor. Currently, in addition to conventional radiotherapy and chemotherapy treatments, various combination immunotherapy regimens have been applied clinically,^[16,17]^ as they are able to mediate better antitumor effects compared to monotherapy.^[18]^ It is also extremely important currently to explore the molecular pathways driving tumor progression in LUAD and to seek new targets.

ELOVL6, the 6th member of its family of genes, specifically catalyzes the prolongation of C12, C14, C16 saturated or monounsaturated fatty acids and plays a key role in the conversion of palmitic acid (C16:0) to stearic acid (C18:0).^[19]^ QSA et al analyzed the plasma metabolic profile of lung cancer patients and indicated that palmitic acid (C16:0) was the one of the most effective metabolites for identifying the stage and type of lung cancer.^[20]^ Endogenous fatty acids are associated with the growth and proliferation of tumor cells, with palmitic acid (C16:0) playing a major role.^[21]^ In this study, we explored the expression of ELOVL6 in LUAD versus normal tissues based on bioinformatics, and the results showed that ELOVL6 expression was significantly upregulated in LUAD, and high ELOVL6 expression was associated with reduced survival in LUAD patients and could be an independent prognostic factor for LUAD patients.

It is well known that the factors affecting the prognosis of tumor patients are tumor cell invasiveness, tumor type and stage. In this study, we tried to identify the co-expressed genes associated with ELOVL6 and explored the potential mechanism and function of ELOVL6 involved in LUAD, and found that the high expression of co-expressed genes, such as GPR87, PAEP, TCN1, VSIG1, ANLN, MYBL2, UBE2C, EREG, and SLC2A1 was negatively correlated with the prognosis of LUAD patients. It has been found that inhibition of the expression of GPR87 and ANLN in LUAD cells reduces the expression of Vimentin and N-cadherin and increases the expression of E-cadherin in the cells, thus reducing the invasion and migration of LUAD cells.^[22,23]^ ANLN controls the metastasis of LUAD cells through the PI3K/Akt signaling pathway.^[24,25]^ TCN1 plays multiple roles in maintaining the basic functions of cell proliferation and metabolism, and its high expression correlates with tumor aggressiveness and poor prognosis.^[26,27]^ MYBL2 acts as an oncogenic transcription factor, which promotes the proliferation and migration of lung cancer cells through the up-regulation of NCAPH.^[28,29]^ What more, some researchers found that the subpopulation of cancer cells expressing UBE2C increased during the invasion of lung adenocarcinoma cells into normal tissues, distributed in the periphery of cancerous tissues, and possessed strong invasiveness.^[30]^ And the expression level of UBE2C gradually increases as the degree of tumor differentiation decreases.^[31]^ The emergence of drug resistance during the treatment of tumors often indicates a poorer prognosis for patients. Tumor-associated macrophage interaction through the EREG/EFGR pathway induces resistance to tyrosine kinase inhibitors in patients with LAUD,^[32]^ while EGFR mutations have been found to lead to EREG overexpression through activation of the MEK/ERK pathway,^[33]^ whereas 30% to 50% of patients with lung adenocarcinoma possessed mutations in the EGFR gene. SLC2A1 is associated with activated CD4 + cells, memory T cells, and poor subsequent immunotherapy.^[34]^

It is well known that immune cells in the LUAD tumor microenvironment (TME) are closely related to the survival prognosis of patients and the therapeutic targets of drugs.^[35]^ Resting regulatory T cells in the TME have weak immunosuppressive activity but differentiate into activated regulatory T cells with strong immunosuppressive activity after stimulation by T cell receptors. Regulatory T cells with strong immunosuppressive function have been shown to infiltrate heavily into various cancer tissues including LUAD.^[36,37]^ Regulatory T cells undergo immunosuppression within TME through activation of STAT signaling molecules.^[38,39]^ They can also competitively bind IL-2,^[40]^ secrete TGF-β and IL-10^[41]^ thereby inhibiting CD8 + T cell activation, proliferation, and the ability to kill tumors. Myeloid-derived suppressor cells can exert immunosuppressive effects by inhibiting the activation of STAT5 signaling pathway, leading to downregulation of MHC -II class molecule expression and causing T cell apoptosis.^[42,43]^ B cells have their unique antitumor immune role in LUAD. It was found that B-cell proliferation was observed in approximately 35% of lung cancers, and tumor-infiltrating B lymphocytes were involved in humoral and cellular immunity and behaved differently between histological subtypes and were observed in all stages of lung cancer,^[44–46]^ suggesting that B cells play a key role in lung cancer progression. The results of the present study suggest that ELOVL6 expression is positively correlated with tumor-infiltrating regulatory T cells and myeloid-derived suppressor cells expression and negatively correlated with B cell expression, indicating that ELOVL6 may be involved in tumor immune escape and suppression of antitumor response through regulation of TME.

In the treatment of LUAD, drug-targeting studies including epidermal growth factor receptor tyrosine kinase inhibitors (EGFR-TKIs) have made great progress, but the development of acquired resistance often leads to treatment failure and disease progression. Cell Miner database-based analysis showed that high ELOVL6 expression was negatively correlated with drug sensitivity of EGFR-TKIs such as Lapatinib and Afatinib. It has been shown that EGFR-TKIs can acquire drug resistance through T790M mutation, Met amplification, and HER2 amplification.^[47]^ Among them, Her2 amplification and MET amplification can trigger the abnormal function of various downstream signaling pathways, including PI3K-AKT, and promote cell transformation, cell invasion, cell proliferation, and cell cycle progression,^[48,49]^ and the GSEA analysis in this study indeed also showed that ELOVL6 was enriched in the PI3K-AKT signaling pathway. Meanwhile, some studies have shown that during platinum-based chemotherapy, tumor cells are resistant to platinum through the PI3K-AKT pathway, which results in the overexpression of anti-apoptotic proteins and defective mitochondrial signaling.^[50,51]^ P53 signaling pathway can also act on mitochondria to cause apoptosis and cellular senescence,^[52]^ and if the P53 signaling pathway is abnormal, it will lead to the If the P53 signaling pathway is abnormal, it will lead to failure of checkpoint response, cell cycle arrest, and apoptosis,^[53]^ which ultimately leads to the poor effect of platinum-based chemotherapy. The E2F signaling pathway and the G2M checkpoint are also involved in platinum resistance.^[54,55]^ In this study, GSEA analysis showed that ELOVL6 is involved in the E2F signaling pathway, G2M checkpoint, MTORC1 signaling pathway, apoptosis, PI3K-AKT-MTOR signaling pathway and P53 signaling pathway, suggesting that ELOVL6 may play an important role in regulating various cellular functions such as cell cycle, proliferation, metabolism, and survival.^[56–59]^ Among them, the E2F signaling pathway, G2M checkpoint, PI3K-AKT-MTOR signaling pathway and P53 signaling pathway are involved in tumor resistance to EGFR-TKI and platinum-based drugs, respectively.^[54,55,60–63]^ This suggests that up-regulation of ELOVL6 may mediate the development of associated drug resistance through the signaling pathways described above.

We found that the expression level of ELOVL6 was significantly higher in LUAD tissues than in normal lung tissues by immunohistochemistry of several LUAD tissues (Fig. 1D and E), and the OS of LUDA patients in the ELOVL6 low expression group was significantly longer than that of patients in the ELOVL6 high expression group. In addition, the results of univariate and multifactorial analyses showed that LUAD was an independent factor in the prognosis of HCC patients.

As shown above, ELOVL6 expression plays an important role in the course of LUAD, and the expression level of this gene has a close relationship with clinicopathological characteristics and survival prognosis, with the possibility of becoming a prognostic marker and therapeutic target for LUAD. Meanwhile, there was a statistically significant correlation between ELOVL6 expression abundance and tumor immune infiltration level, which may provide new ideas for LUAD treatment.

## Author contributions

**Conceptualization:** Bin-Yu Chen.

**Data curation:** Bin-Yu Chen, Kaiyu Shen.

**Formal analysis:** Bin-Yu Chen, Kaiyu Shen, Tian tian Zhang.

**Supervision:** Wen-cang Gao.

**Writing – original draft:** Bin-Yu Chen, Kaiyu Shen.
